# Preparation and investigation of a novel combination of *Solanum nigrum-*loaded, arabinoxylan-cross-linked β-cyclodextrin nanosponges for the treatment of cancer: *in vitro*, *in vivo*, and *in silico* evaluation

**DOI:** 10.3389/fphar.2023.1325498

**Published:** 2023-12-06

**Authors:** Hamid Saeed Shah, Sumera Zaib, Imtiaz Khan, Mahmoud A. Sliem, Osama Alharbi, Mohammed Al-Ghorbani, Zobia Jawad, Kiran Shahzadi, Sajjad Awan

**Affiliations:** ^1^ Institute of Pharmaceutical Sciences, University of Veterinary and Animal Sciences, Lahore, Pakistan; ^2^ Department of Basic and Applied Chemistry, Faculty of Science and Technology, University of Central Punjab, Lahore, Pakistan; ^3^ Department of Chemistry and Manchester Institute of Biotechnology, The University of Manchester, Manchester, United Kingdom; ^4^ Department of Chemistry, Faculty of Science, Taibah University, Medinah, Saudi Arabia; ^5^ Ladywillingdon Hospital, King Edward Medical University, Lahore, Pakistan; ^6^ College of Pharmacy, University of Sargodha, Sargodha, Pakistan

**Keywords:** β-cyclodextrin, cancer, flow cytometry, nanosponges, *Solanum nigrum*

## Abstract

**Introduction:** Cancer contributes to a high mortality rate worldwide spanning its diversity from genetics to resistant therapeutic response. To date emerging strategies to combat and manage cancer are particularly focused on the development of targeted therapies as conventional treatments account for the destruction of normal cells as well. In this regard, medicinal plant-based therapies are quite promising in imposing minimal side effects; however, limitations like poor bioavailability and stability of bioactive phytochemicals are associated with them. In parallel, nanotechnology provides nominal solution to deliver particular therapeutic agent without compromising its stability.

**Methods:** In this study, *Solanum nigrum*, an effective medicinal plant, loaded arabinoxylan cross-linked β-cyclodextrin nanosponges (SN-AXCDNS) were designed to evaluate antitumor activity against breast cancer. Therefore, SN-AXCDNS were prepared by using cross-linker melt method and characterized by physicochemical and pharmacological parameters.

**Results:** Hydrodynamic size, zeta potential and entrapment efficiency (EE%) were estimated as 226 ± 4 nm, −29.15 ± 5.71 mV and 93%, respectively. Surface morphology of nanocomposites showed spherical, smooth, and porous form. Antitumor pharmacological characterization showed that SN loaded nanosponge demonstrated higher cytotoxicity (22.67 ± 6.11 μg/mL), by inducing DNA damage as compared to void SN extract. Flow cytometry analysis reported that encapsulated extract promoted cell cycle arrest at sub-G1 (9.51%). Moreover, *in vivo* analysis demonstrates the reduction in tumor weight and 85% survival chances in nanosponge treated mice featuring its effectiveness. In addition, *in silico* analysis revealed that β-cyclodextrin potentially inhibits MELK in breast cancer cell lines (B.E = −10.1 Kcal/mol).

**Conclusion:** Therefore, findings of current study elucidated the therapeutic potential of β-cyclodextrin based nanosponges to be an alternative approach regarding the delivery and solubilization of antitumor drugs.

## Graphical Abstract

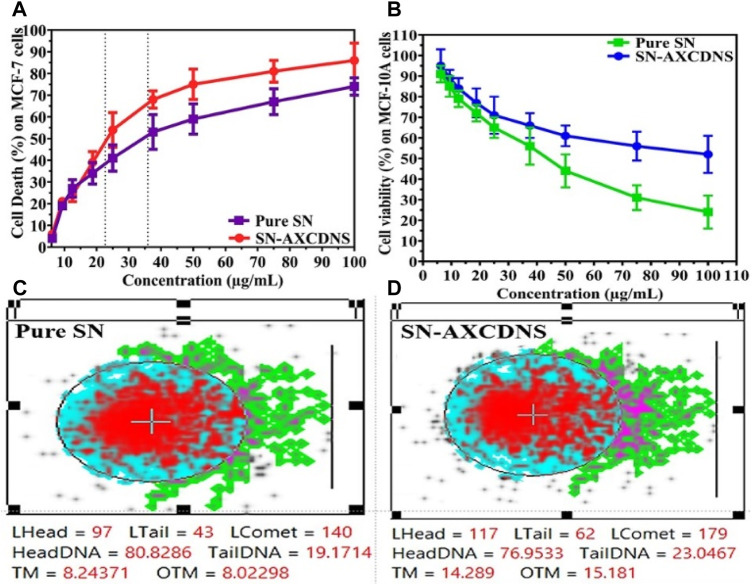

## 1 Introduction

Cancer has been a common health challenge to human beings since decades and is characterized by abnormal growth and proliferation of cells in benign and metastatic forms. According to GLOBOCAN 2020 estimates, approximately 19.3 million new cases and 10 million deaths were reported due to cancer globally (Deo et al., 2022). Worldwide cancer burden has been increasing day by day, accounting for current epidemiological and demographic transitions. Genotypically, cancerous cells demonstrate seven types of alteration in cell physiology, leading to their abnormal proliferation (Fouad and Aanei, 2017). To date, emerging strategies to combat and manage cancer are particularly focused on the development of targeted therapies, as conventional treatments, including chemotherapy, radiotherapy, and surgery, account for the destruction of normal cells, leading to several complications afterward (Albano et al., 2021). Plant-derived chemicals are a significant source of anticancer agents and induce minimal side effects. Medicinal plants have been considered an alternative approach since decades due to their chemoprotective and anticancer potential (Gezici and Şekeroğlu, 2019).

*Solanum nigrum* L. (Mako) (SN), belonging to the family Solanaceae, is an annual herb characteristically branched and smooth, reaching up to 1 m height. It is found in Asia, Africa, and particularly in arable lands. Categorically, it has two varieties based on the fruit it bears: one type grows black fruit and second bears red fruit. In the Indian traditional medicinal system, it is one of the most used herbs as its various parts, such as leaves, fruits, and dried flowers, are used for the treatment of a variety of ailments, such as rheumatism, fever, and hepatitis*.* Several common phytochemicals reported to be responsible for their biological activities include glycosides, alkaloids, flavonoids, saponins, carbohydrate, coumarins, and phytosterol. Some bioactive compounds, such as uttroside B, solanine, solamargine, and physalins, are reported to show anticancer potential against hepatocellular carcinoma (Sekaran et al., 2020), prostate cancer (Nawaz et al., 2021), ovarian cancer (Palanisamy et al., 2021), breast cancer, and lung cancer (Churiyah et al., 2020). Such bioactive compounds are not efficient enough to directly reach the pinpoint target site in the cancerous cell and also do not possess enough penetration capability. Thus, to overcome such limitations, researchers introduced nanocarrier systems which are designed to carry the drug to the targeted location (Thomas et al., 2022).

Nanotechnology has a wide range of applications in the field of medicine and nutraceuticals, characterized in different forms such as nanoparticles, nanotubes, nanofibers, and nanocomposites (Chamundeeswari et al., 2019). Nanosponge (NS) is one of the cutting-edge nanocarriers, characterized as compact, cross-linked polymeric porous structures (Bolmal et al., 2013). NSs function as transporting catalysts and gases and halt the activity of enzymes and adsorption of toxic compounds. They demonstrate high biodegradability, efficient stability over temperature and pH fluctuations, non-cytotoxicity, and effective biocompatibility (Allahyari et al., 2021). Different types of nanosponges have been reported, including metallic, β-cyclodextrin (CD), silicon-based, ethylcellulose, and DNAzymes (Tiwari and Bhattacharya, 2022). Generally, CDNS is the most studied NS due to its selectivity and specificity toward the delivering agent, extraordinary 3D cross-linked network, insignificant toxicity, and controlled release capability ((Lembo et al., 2018). They are generally the enzymatic product of starch, linked together in a ring network, and comprised of glucopyranose units linked by an α-(1,4) glycosidic bond. Commonly, at least 6–8 cyclic glucose units are present in CD, such as α-cyclodextrin, β-cyclodextrin, and γ-cyclodextrin consisting of six, seven, and eight units, respectively (Fink, 2021). Due to the presence of a hydroxyl group, it can be directly co-polymerized with different types of monomers, thus imparting reactivity. The surface area and porosity of nanosponges are affected by the cross-linked polymers, as an increased cross-linked polymer amount on the surface allows the formation of a small-sized nanosponge with effective porosity (Asela et al., 2021). Different modifications have also been carried out on the surface of NS, such as introducing functionalized cholesterol in cyclodextrin-based NS, making it disperse in the cell and function to bind with different proteins. After the surface functionalization, the desired drug loaded in the nanosponge improves bioavailability. Enhanced antitumor activity by doxorubicin (Dox)-loaded glutathione-responsive cyclodextrin has also been reported (Daga et al., 2020).

In view of the literature findings, the current study aims to synthesize *S. nigrum*-loaded arabinoxylan (AX)-cross-linked β-cyclodextrin nanosponges for antitumor activity. The *in vitro* results were further complemented with *in vivo* testing and *in silico* modeling.

## 2 Materials and methods

### 2.1 Preparation of the SN extract

The plant material was thoroughly washed with distilled water to remove dust and impurities and dried for 12 days under shade. The material was finely powdered using a mechanical grinder and then sieved. A measure of 5 g of powder was added to 300 mL of distilled water to prepare the aqueous extract. The mixture was boiled for 10 min, and the watery extract was filtered after 24 h (Mousavi-Kouhi et al., 2022).

### 2.2 Extraction of arabinoxylan from ispaghula seeds

Collected seeds (1 g) were washed with water and soaked in 200 mL boiling distilled water until a thick gel was formed. As the mixture settled down, AX was separated and dried in an oven at 37°C for 3 days to collect the powder (Iqbal et al., 2011).

### 2.3 Synthesis of β-cyclodextrin nanosponges via the melt method

Cyclodextrin nanosponges were prepared using diphenyl carbonate (DPC) as a cross-linker (Pushpalatha et al., 2018). A measure of 2 g of diphenyl carbonate (0.0093 M) was melted at 90°C, and 1 g of cyclodextrin (0.00088 M) was added to it and allowed to react for 5 h. The resultant product was washed with water and Soxhlet-extracted with acetone for 24 h to remove unreacted ingredients. The purified DPC-CDNSs were dried at 60°C for 24 h and stored at an ambient temperature until further use. Three types of DPC-CDNSs were prepared using a cross-linker at different molar ratios with respect to cyclodextrin, namely, 1:2, 1:4, and 1:6 (CD:DPC).

### 2.4 Loading of drugs onto NS

In order to prevent the development of aggregates, the NS was suspended in water and sonicated for few minutes. The drug combination was disseminated in excess in the aqueous solution. To permit complexation between the CDNS and drug, the resulting suspension was kept under continuous stirring for 5 h. Subsequently, the uncomplexed drug was separated from the complexed drug by centrifugation at 2,000 rpm for 10 min. The recovered silt was freeze-dried to form a solid powder of drug-loaded NS (Erum et al., 2014; Petitjean et al., 2020; Gangwar and Akhtar, 2022).

### 2.5 Physical characterization of SN-AXCDNS

#### 2.5.1 Entrapment efficiency

Entrapment efficacy was measured using a previously reported method (Pushpalatha et al., 2018). SN-AXCDNS formulation (10 mg) was added to 10 mL of PBS under magnetic stirring for 1 h at 100 rpm and injected into the dialysis membrane submerged into 500 mL PBS (7.4 pH). Absorbance was measured using a UV-visible spectrophotometer at 426 nm. The EE was calculated using the following formula:

$$\text{EE}\%=\left(\text{SN entrapped in AXCDNS}/SN\;added\;in\;AXCDNS\right)\times 100.$$

#### 2.5.2 Surface morphology of nanocomposites

The surface morphology of nanocomposites was evaluated using the Hitachi S-4700 scanning electron microscope (SEM), with an acceleration voltage ranging from 10 to 20 kV. The SN-AXCDNSs were dispersed using ethanol and promptly deposited onto pristine silicon wafers. To facilitate conduction, the samples were covered with a layer of gold by sputter-coating.

#### 2.5.3 Hydrodynamic diameter and zeta potential estimation

To estimate the hydrodynamic diameter of the formulated SN-AXCDNS, the dynamic light scattering (DLS) method was used, and SN-AXCDNS was dispersed in double distilled water. The zeta potential was measured using a Zetasizer Nano ZS instrument.

#### 2.5.4 Drug release kinetics

To simulate the *in vivo* environment, SN-AXCDNS (equivalent to 10 mg SN) was diluted in 10 mL of PBS with pH 7.4, shifted to a dialysis bag immersed in 500 mL of PBS containing 1.2 μg/mL of lysozyme, and agitated using a magnetic stirrer (75 rpm, 37°C). The release of SN from NS was assessed at a wavelength of 426 nm using a spectrophotometer. In addition, the release kinetic model was examined using the DDSolver tool to investigate the primary release mechanism.

### 2.6 Pharmacological characterization

#### 2.6.1 Sulforhodamine B assay

The sulforhodamine B (SRB) assay was used to investigate the anti-proliferative activity of pure SN extract and SN-loaded AXCDNS over the MCF-7 cell line, while the cytotoxicity toward normal cells was estimated by treating non-cancerous MCF-10A cells (Vichai and Kirtikara, 2006). Cells (1 × 10^4^) were grown for 24 h in a 96-well plate. The developed cells were treated with different concentrations of pure SN extract and SN-loaded AXCDNS and incubated for 24 h. Ice-cold 40% trichloroacetic acid (TCA) was used to fix the cells. The cells were washed with PBS and placed in the open air for drying. SRB dye (0.4% w/v) was used to stain the cells for 30 min. Afterward, 10 mM (pH 10.5) of 100 µL Tris-base was used to swirl the cells. The readings were taken on an ELISA microplate reader at 565 nm. IC_50_ (µg/mL) was calculated using GraphPad Prism 5.0.

#### 2.6.2 Genotoxicity assessment (comet assay)

To investigate genotoxicity or to evaluate the DNA double-strand break, the comet assay was performed using a previously developed method (Singh et al., 1988; Priyadarsini et al., 2010). MCF-7 cell suspensions (2 × 10^4^ cells/well) were treated with pure SN extract and SN-AXCDNS, placed over a comet slide, and supplemented with 1% LMPA. The slides were immersed in lysis solution (10 mM Trizma-X, 10% DMSO, 2.5 M sodium hydroxide, 1% Triton-X, and 100 mM of EDTA with pH 10). The samples were kept in a horizontal electrophoresis tank containing 300 mM of NAOH and 1 mM EDTA with pH 13 for the time-course experiment. In this alkaline pH, DNA unwound itself. The comet slides were washed with methanol and dried. DNA damage was investigated using CaspLab 1.2.3b2 tools.

#### 2.6.3 Flow cytometry analysis

To conduct flow cytometry analysis, MCF-7 cells were (1 × 10^4^) incubated for 24 h with pure SN extract and SN-AXCDNS. Trypsinization was performed at 37°C for 6 min using trypsin and EDTA solution. Media were added gradually to prevent clumping of cells. Cells were treated with H_2_O_2_ and placed in the binding buffer (100 µL) for 15 min and then in the dark for 15 min after treating with annexin-V FITC dye and propidium iodide (PI). Fluorescence-activated cell sorting (FACS) with the respective filter of wavelength for annexin-V FITC (600 nm) and PI (545 nm) was used to analyze cells. Approximately 10,000 cells in one cycle were assessed using the CytoFLEX instrument. The results were represented in the cell cycle histogram plot.

#### 2.6.4 Animal studies

Albino female adult BALB/C mice (25–30 g) were retained at the Faculty of Science and Technology, University of Central Punjab, Lahore, Pakistan. Mice were provided with unfettered feed and water and kept for 12 h under a 12 h light/dark cycle. Five albino mice were placed in a steel mesh cage to avoid discomfort by overcrowding. The animals were treated in accordance with the guiding principles set by the Institution’s Ethics Committee on the treatment of animals in research at the University of Central Punjab, Lahore. Mice were divided into five groups containing five albino mice each. A measure of 100 μL of 4 × 10^6^ MCF-7 cells was injected into selected groups (groups 1–4). Over a period of 20 days, the tumor was allowed to grow up to 50 mm^3^ (Guo et al., 2011). Each group, along with its respective treatment chart, is given in Table 1.

**TABLE 1:** Anticancer effects on both experimental and control groups of mice treated with pure SN extract, SN-AXCDNS, and cisplatin.

Group	Type of treatment
1	Without treatment
2	Cancerous mice were given 3 mg/kg cisplatin
3	Pure SN extract (64.63 mg/kg)-treated cancerous mice
4	Pure SN extract- and SN-AXCDNS (40.8 mg/kg)-treated cancerous mice
5	AXCDNS (40.8 mg/kg)-treated non-cancerous mice

The tumor inhibition rate (TIR) was calculated to comprehend how well each formulation functioned against cancer.

$$\text{TIR}\left(\%\right)=\left[\left(\text{Tumor weight of sample group}\right)/\left(\text{Tumor weight of control group}\right)\right]\times 100.$$

### 2.7 Statistical analysis

Statistical analysis was conducted using the paired *t*-test for flow cytometry and genotoxicity experiments. A significance degree of 95% (*p* < 0.05) was set. Microsoft Excel 2010 and Prism 5.0 and SPSS 9.0 software were used. The reported results were expressed as mean standard deviation (SD).

### 2.8 *In silico* analysis

For analyzing the anticancer activity of β-cyclodextrin computationally, it is crucial to predict its potential targets. As the anticancer activity of β-cyclodextrin has been evaluated on MCF-7 cancer cell lines, three essential target proteins, namely, Aurora kinase, maternal embryonic leucine zipper kinase (MELK), and T-lymphokine-activated killer cell-originated protein kinase (TOPK), which are highly expressed in breast cancer cells, were investigated [29] (Yoshimaru et al., 2021). Molecular docking was performed using AutoDock Vina to examine the interaction between β-cyclodextrin and all three target proteins. The three-dimensional structures of target proteins, with PDB IDs 5EW9 (Aurora kinase) (De Groot et al., 2015), 5TWL (MELK) (Huang et al., 2017), and 5j0a (TOPK) (Dong et al., 2016), were obtained from the Protein Data Bank (PDB). The selected 3D Aurora kinase consists of 271 amino acid residues and shows no mutations with a resolution of 2.18 Å (De Groot et al., 2015). The MELK protein (5TWL) is made up of 341 amino acid residues having a resolution of 2.42 Å, with no mutation reported in the structure (Huang et al., 2017). Similarly, 300 amino acid residues are present in TOPK, having a sole reported mutation and a resolution of 2.74 Å (Dong et al., 2016).

Prior to docking in AutoDock Vina 1.1.2, the ligand and target proteins were prepared using BIOVIA Discovery Studio 2021 molecular visualization software. The grid for each of the target proteins was adjusted separately using AutoDock Tools 1.5.7, and docking was performed using AutoDock Vina 1.1.2 (Al-Ghani et al., 2022). Subsequently, the intermolecular interactions between β-cyclodextrin and amino acid residues in the binding pocket of each of the target protein were visualized using Discovery Studio 2021 molecular visualization software (Kumar et al., 2020).

## 3 Results and discussion

### 3.1 Physical characterization of SN-AXCDNS

#### 3.1.1 Hydrodynamic and zeta potential estimation

The hydrodynamic size of SN-AXCDNS was estimated to be 226 ± 4 nm (Table 2). The diameter of NS is a significant parameter to estimate its performance regarding drug release and adsorption. Small-sized nanoparticles facilitate an efficient drug release mechanism. The normal size of NS ranged from 234.95 to 374.26 nm (Aggarwal et al., 2016; Gangwar and Akhtar, 2022). Our synthesized SN-AXCDNS is proximally near to the optimal size range. A higher degree of the cross-linked polymer in nano-formulation accounts for the increased diameter. The results of the current study demonstrated that the nanosponge has the capability to aggregate, owing to the structural feature of β-CD to interact with the encapsulating material as reported. After loading the drug into CDNS, the diameter reached up to 100 nm compared to the unloaded NS (Yaşayan et al., 2020). The estimated zeta potential of NS was −29.15 ± 5.71 mV (Table 2).

**TABLE 2:** Physical characterization of SN-AXCDNS.

SN-AXCDNS	Finding
Hydrodynamic diameter	226 ± 4 nm
Entrapment efficiency (%)	93 ± 6
Zero-order	0.9933
First-order	0.9535
Higuchi model	0.8288
Korsmeyer–Peppas model	0.9949
n-value	1.075
Zeta potential	−29.15 ± 5.71 mV

The zeta potential depicted the charge present on the surface of nano-formulation, which accounts for its interaction with the biological system. The presence of charge featured the respective stability of the nanosponge in an aqueous solution (Gangwar and Akhtar, 2022). A highly negatively charged surface of SN-AXCDNS portrayed a lower trend toward agglomeration and exhibited effective stability, which is consistent with a previous study (Gholibegloo et al., 2019). Another study reported that NS with a similar charged surface demonstrated efficient electrostatic stability (Xu, 2008).

#### 3.1.2 Entrapment efficiency

Entrapment efficiency depicts the capacity or trend of a particular nanocarrier to encapsulate a drug, biomolecule, or plant extract (Galvão et al., 2015). Cyclodextrin possesses a ring-like structure comprising exterior hydroxyl edges and a polar hydrophobic interior cavity (da Rocha Neto et al., 2018). The polar cavity of β-CD facilitates the encapsulation of hydrophobic molecules in an aqueous solution (Wang et al., 2014). The standard curve for the SN extract was generated using a UV-visible spectrophotometer (Supplementary Figure S1). The resulting inclusion complex (IC) is dynamic in nature, rendering the release of biomolecules from the cavity. The measured entrapment efficacy of the synthesized SN-AXCDNS is 93% ± 6%, which showed that the CD-based NS exhibited higher entrapment efficacy (EE). The encapsulation capacity of CD-based nanosponges was reported to be affected by intramolecular water moieties, concentration and nature of the loaded material, and nature of the solvent used to prepare the extract (da Rocha Neto et al., 2018). Moreover, the degree of cross-linking also affected the formation of inclusion between the NS and loaded material (Ansari et al., 2011). A previously reported study showed comparable results in which natural polyphenol was encapsulated in CDNS with an EE of 69.17% (Mady and Mohamed Ibrahim, 2018).

#### 3.1.3 Surface morphology of nanocomposites

**FIGURE 1:**
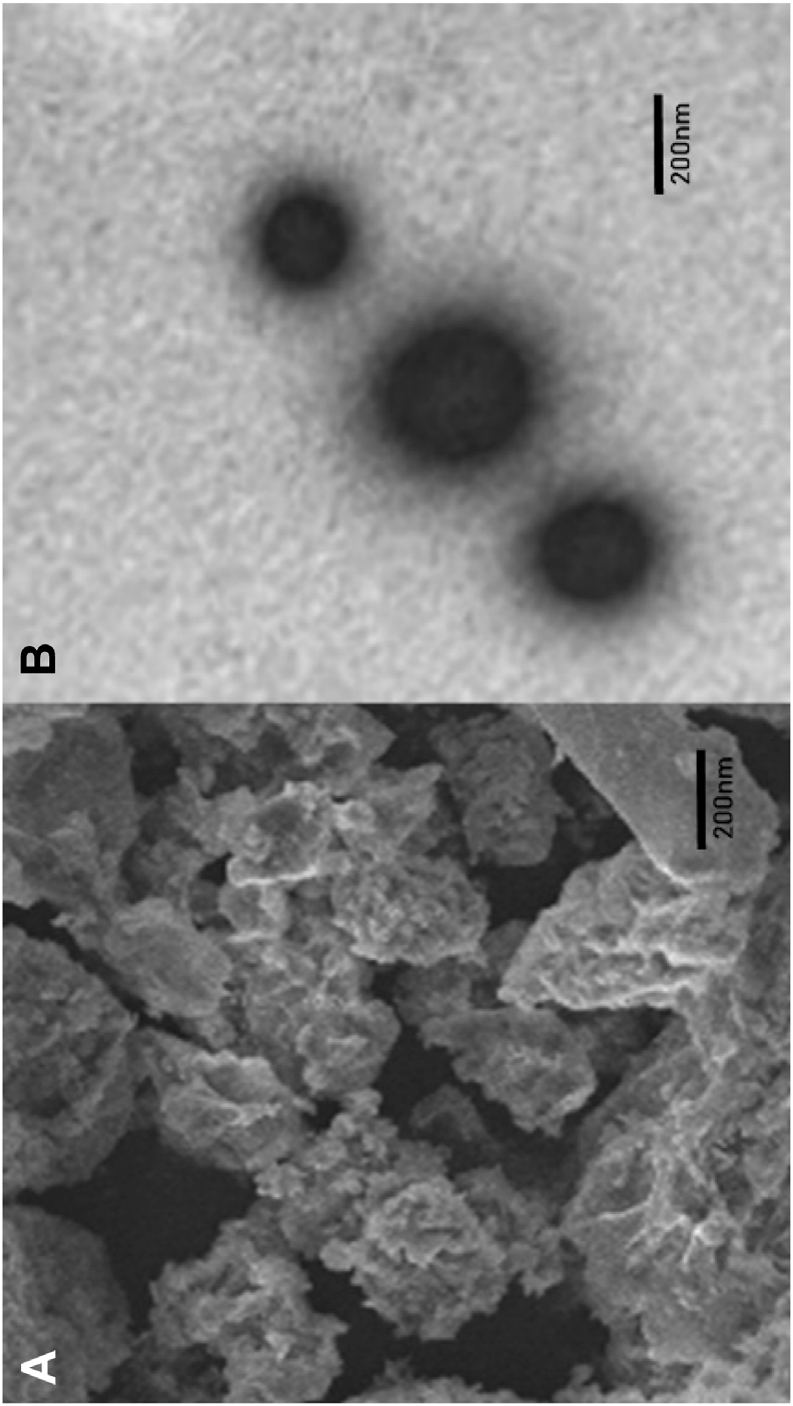
Representation of the surface morphology of SN-AXCDNS by scanning electron microscopy **(A)** and transmission electron microscopy **(B)**.

To determine the surface morphology of SN-AXCDNS, SEM and TEM analyses were conducted. Figure 1A shows the porosity and aggregation of particles, which is consistent with the previous study reporting the porous morphology of CDNS (Varan et al., 2020). Aggregation might be due to the presence of arabinoxylan, as reported in a previous study (De Anda-Flores et al., 2020). It was shown that the formulated nano-form exhibited a spherical smooth morphology under a scale bar of 200 nm, as shown in the photomicrograph of the optimized SN-AXCDNS in Figure 1B. Comparable results were verified in a previous study, which states that the loading of the drug into β-CD-based NS did not affect its spherical morphology (Kumar and Rao, 2021). Another study also described the morphologically spherical CDNS encapsulating babchi oil (Kumar et al., 2018).

#### 3.1.4 Drug release kinetics

**FIGURE 2:**
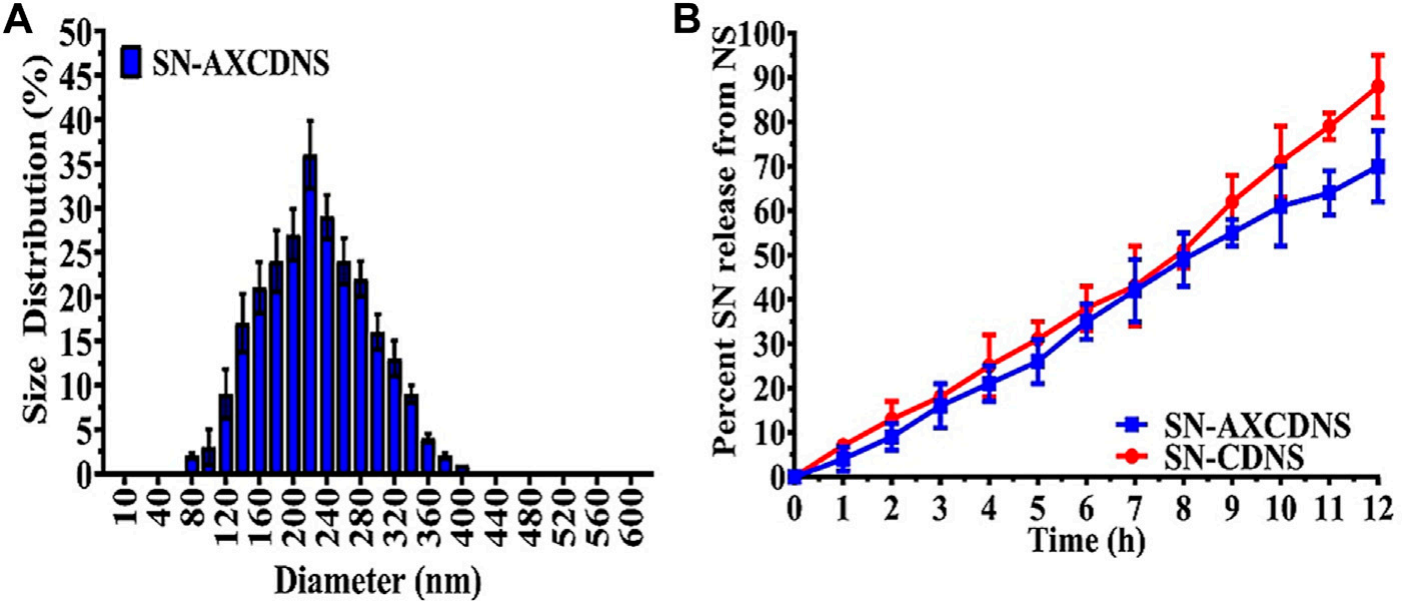
**(A)** Hydrodynamic size distribution of synthesized SN-AXCDNS. **(B)** SN release mechanism from prepared SN-CDNS (red) and SN-AXCDNS (blue).

Figure 2B shows the extract release profile of CDNS and AXCDNS. The results showed that the extract was released in a systemic and regular form. The percentage (%) of release from CDNS and AXCDNS after 12 h was approximately 85% and 65%, respectively. It was reported that the release of the encapsulated material from NS depends on the ratio of the cross-linker (Gholibegloo et al., 2019). The initial burst release was not shown in both cases (CDNS and AXCDNS), and it was stated in studies that anticancerous drugs with a regular release mechanism reduced the chances of toxicity, which are higher in the case of initial burst release (Dora et al., 2016). In order to examine the release kinetic profile of SN, various pharmacokinetic models were used, including zero-order, Higuchi, first-order, and Korsmeyer–Peppas models. The measured regression coefficient (*R* ^2^) values of different models are listed in Table 2. The best fitted model is the zero-order model (*R* ^2^ = 0.9933), which indicated the concentration-independent and slow-release mechanism of SN from the nanosponge. Moreover, a higher value of the Korsmeyer–Peppas model (*R* ^2^ = 0.9949, n = 1.075) depicted non-Fickian anomalous diffusion, an erosion-type release mechanism. More than one release mechanism of CDNS was also stated in a previous study (*R* ^2^ = 0.991) (Shringirishi et al., 2017).

### 3.2 Pharmacological characterization

#### 3.2.1 Anticancer activity

**FIGURE 3:**
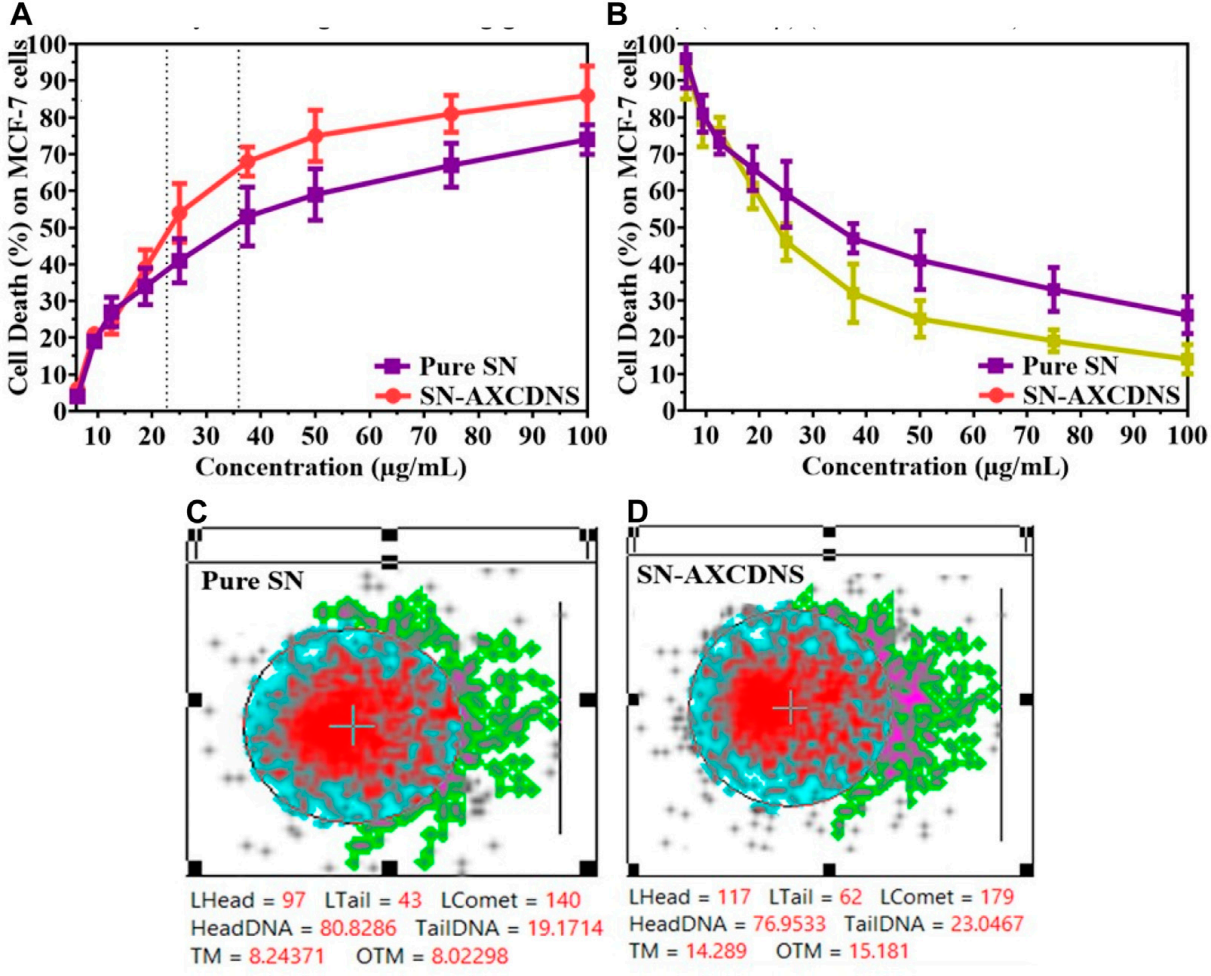
The pharmacological description evaluates the percentage of cell death **(A)** and cell viability **(B)** induced by pure SN extract and SN-AXCDNS on MCF-7 and MCF-10A cells, respectively. DNA impairment in the MCF-7 cell line exposed to pure SN extract **(C)** and SN-AXCDNS **(D)**.

To screen the therapeutic antitumor or anti-proliferative potential of the synthesized nanosponge (SN-AXCDNS) against the breast cancer cell line (MCF-7), the SRB assay was performed. Principally, the SRB assay was executed to evaluate the cytotoxicity and cellular proliferative potential of particular synthetic or natural drugs. The protein content of TCA-fixed cells binds with the SRB dye characterized as bright pink dye having sulfonic groups which have the affinity to bind with basic amino acids under a strong acidic condition (Shakil et al., 2022). It provides the concentration-dependent estimation, representing the quantity of protein in cells (Orellana and Kasinski, 2016). In this study, estimated IC_50_ for pure SN extract was calculated to be 35.91 ± 4.63 μg/mL, and for encapsulated (SN-AXCDNS), it was 22.67 ± 6.11 μg/mL, as shown in Figure 3A. Cell viability was assessed in MCF-10A epithelial breast cells to ascertain the potential toxicity of pure SN extract and SN-AXCDNS across various concentrations (Figure 3B).

Lowest IC_50_ represented that SN-AXCDNS exhibited higher anticancer activity, while the pure extract exhibited a higher IC_50_ value, depicting relatively less activity. This might be due to the increased solubility of the extract as reported previously that the nanosponge enhanced the solubility and oral bioavailability of the encapsulated material. Previously, antitumor activity of the SN extract was also reported against high-grade gliomas, bladder cancer, non-small-cell lung cancer, hepatocellular carcinoma, cervical cancer, colorectal carcinoma, and breast cancer. It was reported that several bioactive compounds in the extract were responsible for its cytotoxic activity, such as steroidal alkaloid, quercetin, and steroidal glycoalkaloids (Shi et al., 2019; Dong et al., 2021; Li et al., 2021; Nawaz et al., 2021; Liu et al., 2022). Several steroidal glycoalkaloids, such as solasdamine, solanidine, solasonine solasodine, solamargine, diosgenin, solavilline, and alpha-solanine, were reported to induce antitumor activity (Ahmad, 2019). Similar results were reported in a previous study in which resveratrol (polyphenol)-encapsulated CD nanosponge induced higher cytotoxic potential in HCPC-I cells than plain resveratrol (Ansari et al., 2011).

#### 3.2.2 Genotoxicity and apoptosis assessment

In eukaryotic cell assessment, the DNA damage was assessed using the comet assay which evaluates the genotoxicity of a particular chemical or natural product to divulge double-strand break (DSB) in the genome. The greater tail intensity obtained in the assay represented a higher frequency of DNA breaks as damaged strands migrated toward the cathode (Langie et al., 2015). To quantify the DNA damage, two known parameters were used, namely, tail moment and olive tail moment (Lu et al., 2017). Figure 3B shows that the tail concentration of DNA in cells treated with SN-AXCDNS was higher compared to the pure SN-treated cells. The results demonstrated that SN-loaded CDNS induced effective genotoxicity in the breast cancer cell line (MCF-7). It was described previously that phenolic contents and terpenoids are the key players in inducing cell death by DNA damage, and there is a higher concentration of terpenoids reported in SN (Paul et al., 2015). Recently, it was reported that degalactotigonin, a steroidal glyoside present in SN, induces antitumor activity through DNA damage, p53 activation, and transforming growth factor-β (TGF-β) inhibition (Li and Gu, 2022).

#### 3.2.3 Flow cytometry analysis

**FIGURE 4:**
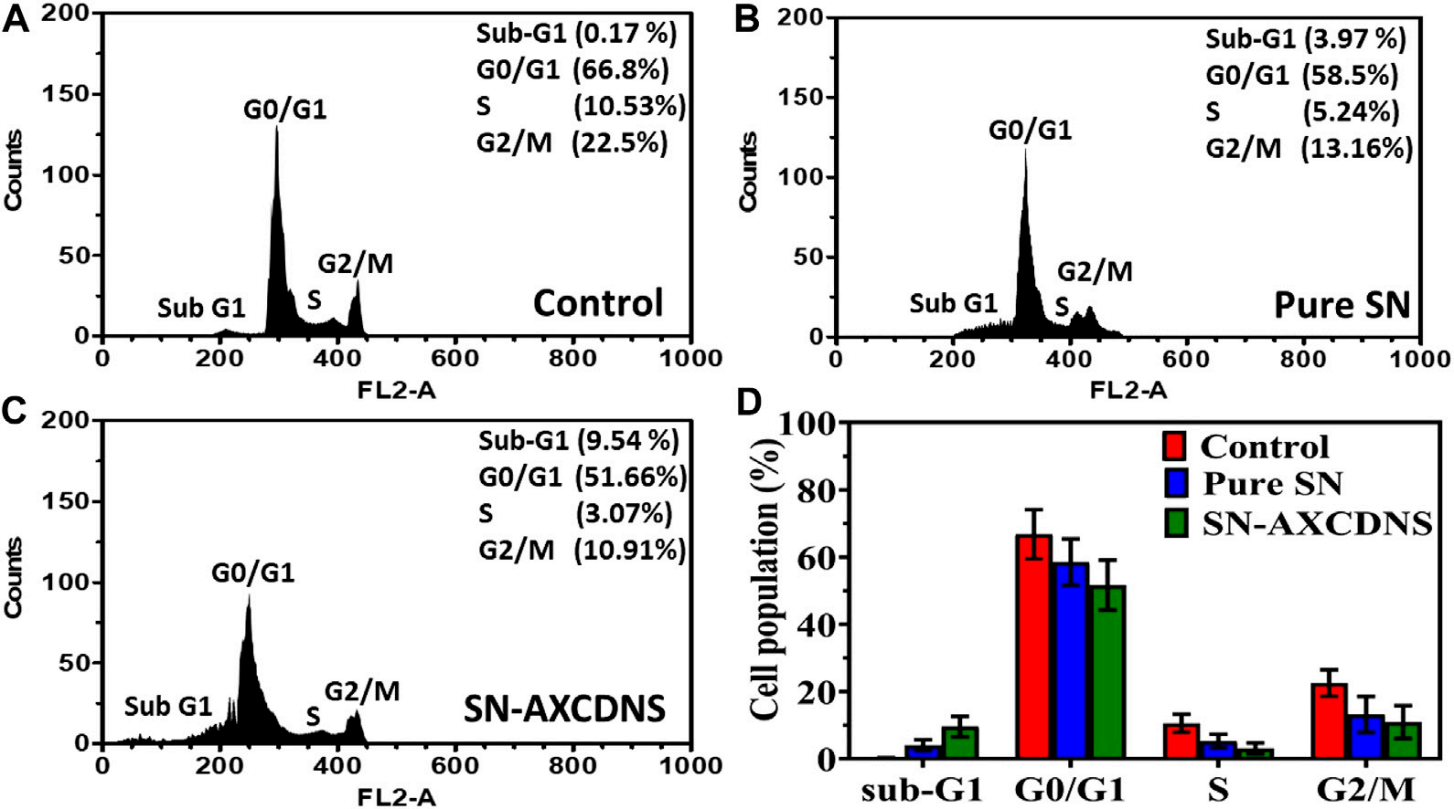
Apoptosis analysis in cell-cycle phases by flow cytometry in the control **(A)**, pure SN extract-treated cells **(B)**, and SN-AXCDNS-treated cells **(C)**. **(D)** The histogram plot shows the % cell population in sub-G1, G0/G1, S, and G2/M phases.

Moreover, cellular death analysis was investigated by flow cytometry, which provides the quantitative analysis of apoptotic bodies in different cell cycle phases (Sub-G1, G0/G1, S, and G2/M), as shown in Figures 4A–D. Histogram analysis validated that the percentage of cells treated with SN-AXCDNS was greater in the sub-G1 phase (9.54%) (Figure 4C) compared to those treated with pure SN extract (3.95%) (Figure 4B) and control (0.17%) (Figure 4A). In the S phase, the percentage of control, pure extract-, and SN-AXCDNS-treated cells was 10.53%, 5.24%, and 3.07%, respectively, representing that NS encapsulating AXCDNS induced higher cell-cycle arrest in the initial phases (G0/G1) of the cell cycle (Figures 4A–D). This might be due to the fact that β-CD nanocarriers facilitate the prolonged exposure of the drug to cancerous cells (Torne et al., 2013). The SN extract is responsible for the cell-cycle arrest in cancerous cells, as reported in a previous study, and degalactotigonin isolated from SN is stated to induce cell-cycle arrest in the G0/G1 phase by inhibiting epidermal growth factor receptors (EGFRs) in PANC1 cells. Furthermore, overexpression of the mRNA level of p21 by degalactotigonin is directed to inhibit the D1/CDK4 complex, mediating cell-cycle arrest in the G1 phase (Tuan Anh et al., 2018). Moreover, the phenolic content of SN was reported to induce sub-G arrest by reducing the level of cell-cycle proteins CDC25A, CDC25B, and CDC25C (Wang et al., 2011).

However, improved efficacy of the plant extract or bioactive components was reportedly achieved by encapsulating within CDNS. It was consistent with a previous study in which a β-CD-encapsulated alkaloid, camptothecin (CPT), induced improved cell-cycle arrest in the S phase compared to the uncapsulated alkaloid in the prostate cancer cell line, due to effective bioavailability (Minelli et al., 2012). The β-CD-encapsulated acetylshikonin (AcSh/β-CD) induced approximately 80% cell-cycle arrest at G0/G1 in HCT-116 and MDA-MB-231 cells, which also supports the effectiveness of cyclodextrin-based nanocarriers (Vukic et al., 2020).

### 3.3 *In vivo* studies

**FIGURE 5:**
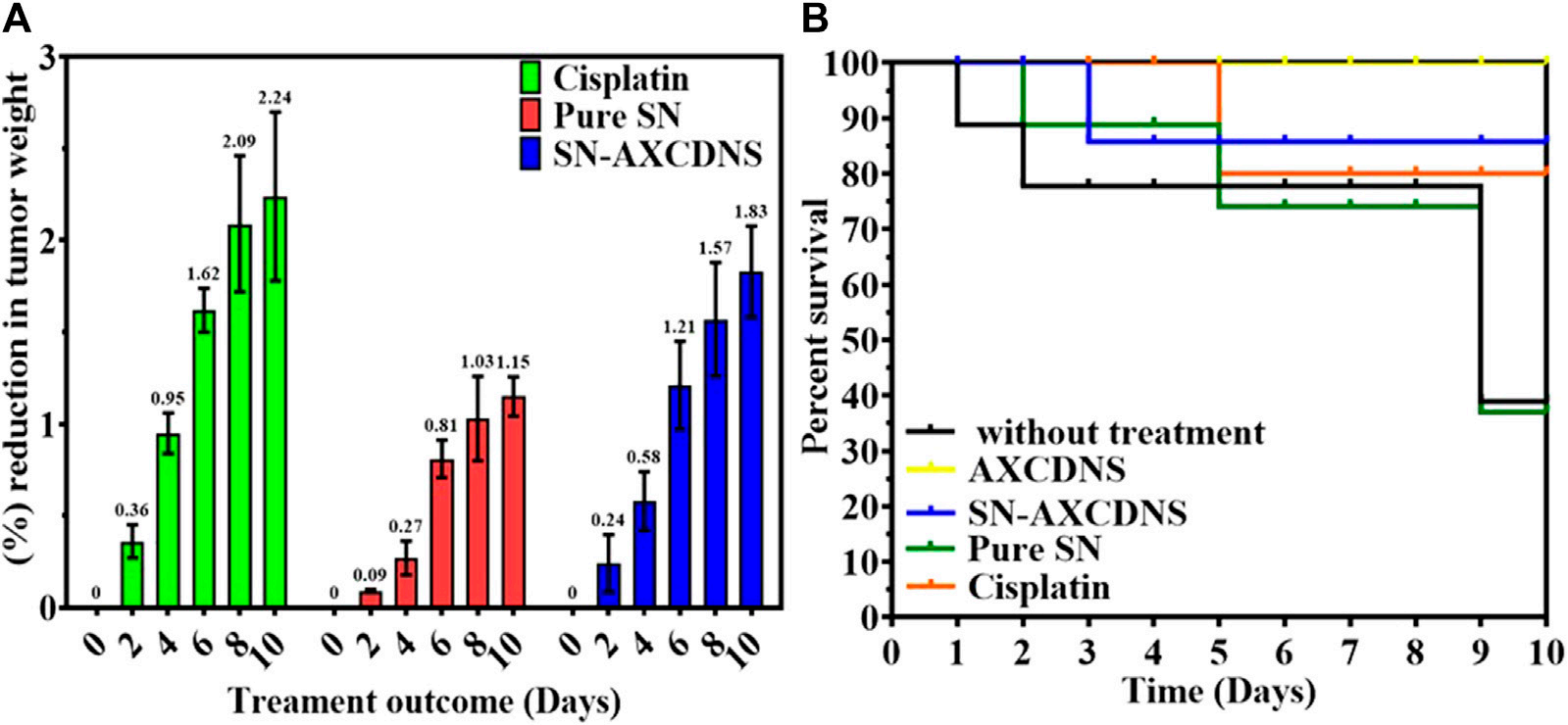
*In* *vivo* analysis. Percent (%) reduction expressed in percentage (%) **(A)** and survival tumor volume **(B)** in control, cisplatin-, pure SN extract-, and SN-AXCDNS-treated mice.

*In vivo* examination was performed over albino female adult mice. Cancer was induced in mice, which were divided into four groups. Groups 2, 3, and 4 were treated with 3 mg/kg cisplatin, 64.63 mg/kg pure SN extract, and 40.8 mg/kg NS-AXCDNS, respectively. The mice in group 1, which were malignant, and group 5, which served as the control group, were not administered any therapy and were provided with AXCDNS (40.8 mg/kg). Tumor reduction and survival percentage were estimated, as shown in Figures 5A, B. The results showed that the tumor weight was reduced to 1.83% in the group treated with SN-AXCDNS and comparable to the group treated with cisplatin (2.24%), while the weight of the tumor was moderately decreased (1.15%) in the group treated with pure SN extract (Figure 5A). Mice treated with SN-AXCDNS exhibited 85% survival chances, which is slightly consistent with the cisplatin-treated group (80%). The group that received the pure SN extract treatment had a survival probability of only 35%, whereas the mice in group 5, which were administered free AXCDNS, displayed a 100% survival rate (Figure 5B).

Previously, the triple-negative breast cancer cell line was treated with docetaxel (a hydrophobic drug)-loaded, hydroxypropyl β-cyclodextrin (DTX/HPCD) nanocarrier, and an enhanced tumor reduction was reported (Ferrati et al., 2015). Another study also reported 60% volume tumor reduction in the breast cancer cell line treated with β-cyclodextrin nanocarriers encapsulating doxorubicin (Argenziano et al., 2020). Free nisin-Z (N-Z), which is an antimicrobial peptide, and cyclodextrin-encapsulated N-Z have been investigated in tumor-induced mice. CD N-Z showed a reduction in tumor weight and volume by mediating apoptosis in cells (Khazaei Monfared et al., 2023). The efficacy of cyclodextrin NS toward reducing the drug-resistant mechanism was investigated previously, in which glutathione-responsive nanosponges were designed to deliver the anticancer drug, doxorubicin, in human HepG2 cells (*in vitro*) and precision-cut liver slices of rats (*in vivo*). *In vivo* analysis showed that Dox-encapsulated GSH-NS demonstrated higher hepatic accumulation compared to free Dox (Daga et al., 2020).

*In vitro* and *in vivo* analyses showed encouraging results. However, *in vivo* data may not necessarily predict similar human clinical outcomes. Therefore, detailed clinical studies are needed to establish the promising efficacy observed in our work. Future research should investigate the potential of AXCDNS for delivering anticancer agents against various cancer types and undertake clinical studies to evaluate their safety and effectiveness in humans.

### 3.4 *In silico* analysis

**FIGURE 6:**
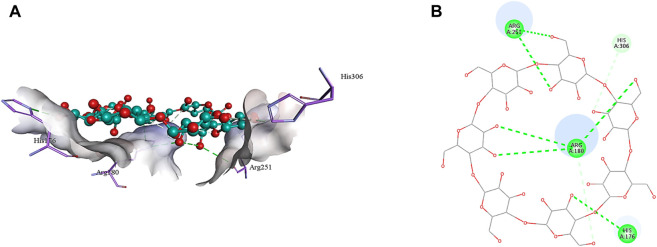
3D **(A)** and 2D **(B)** illustration of intermolecular interactions between Aurora kinase active pocket residues and atoms of β-cyclodextrin.

**FIGURE 7:**
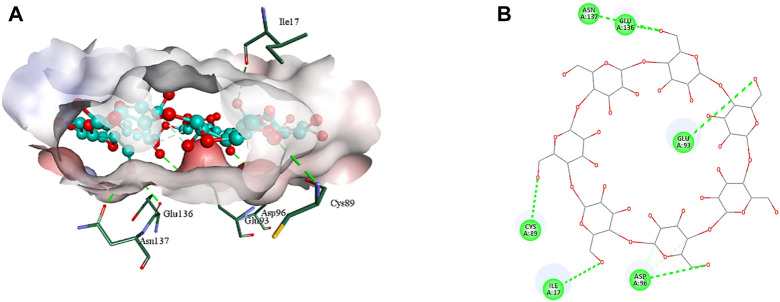
3D **(A)** and 2D **(B)** illustration of intermolecular interactions between MELK active pocket residues and atoms of β-cyclodextrin.

**FIGURE 8:**
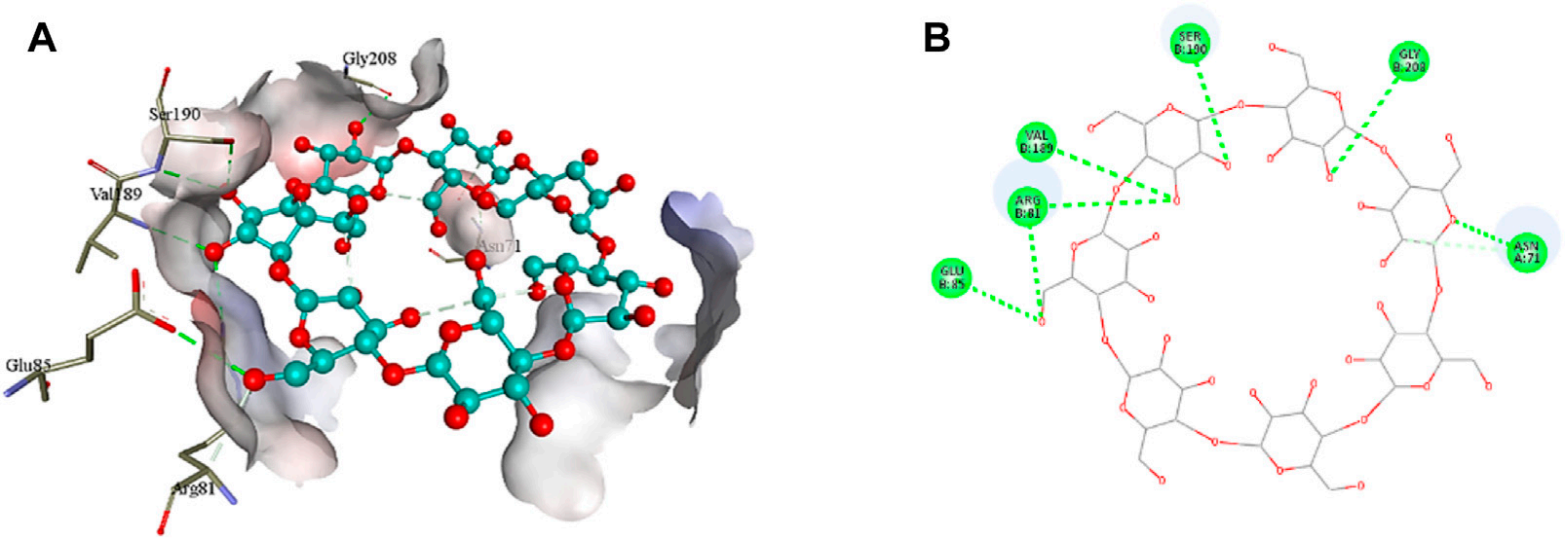
3D **(A)** and 2D **(B)** illustration of intermolecular interactions between TOPK active pocket residues and atoms of β-cyclodextrin.

The 2D structure of β-cyclodextrin was obtained from PubChem (PubChem CID: 444041), and the 3D structures of all the three selected target proteins (Aurora kinase, MELK, and TOPK) were acquired from the Protein Data Bank (PDB) (De Groot et al., 2015; Dong et al., 2016; Huang et al., 2017). The docking was performed using AutoDock Vina 1.1.2, and the results were obtained in the form of binding energies (BEs). At least nine different poses of β-cyclodextrin were generated, and the best pose was selected based on the lowest binding energy. The most effective inhibitory action of β-cyclodextrin was observed when it was docked with the active site of MELK (BE = −10.1 kcal/mol). The binding energy of the Aurora kinase–β-cyclodextrin complex and TOPK–β-cyclodextrin complex was −7.0 and −8.7 kcal/mol, respectively. Afterward, the visualization of protein–ligand intermolecular interactions predicted that β-cyclodextrin interacts with the active site of Aurora kinase by forming a conventional hydrogen bond with His176, Arg180, and Arg251. In addition, His306 of the binding site of Aurora kinase forms a carbon hydrogen bond with O61 of β-cyclodextrin, as shown in Figure 6. Similarly, Ile17, Cys89, Glu93, Asp96, Glu136, and Asn137 of the active site of MELK develop a conventional hydrogen bond with the atoms of β-cyclodextrin within a distance of 5 Å. Asp96 also forms a carbon hydrogen bond with β-cyclodextrin, as shown in Figure 7. Moreover, conventional hydrogen bonds also exist between β-cyclodextrin atoms and residues in the TOPK-binding site, which include Asn71, Arg81, Glu85, Val189, Ser190, and Gly208, as shown in Figure 8.

## 4 Conclusion

In summary, the present study aims to investigate the antitumor potential of arabinoxylan-cross-linked, β-cyclodextrin-encapsulated *S. nigrum* extract against the breast cancer cell line (MCF-7). Nanosponges were reported to act as the cargo for various biomolecules and drugs. In this study, physical and pharmacological characterization of the synthesized NS was evaluated. Morphologically spherical, smooth, and 226-nm-sized nanosponges were synthesized. The SN extract was reported to release in systemic and regular forms. Cytotoxic analysis exposed the improved anticancerous potential of the SN extract, which featured higher bioavailability and stability in the target site. Genotoxicity assessment revealed that higher DNA damage was achieved by SN-AXCDNS compared to void SN extract, which induced effective genotoxicity. Quantitative analysis of apoptosis was performed by flow cytometry, and the results reported that SN-AXCDNS induced cell-cycle arrest at sub-G1 and S phases. Furthermore, *in vivo* antitumor activity showed that the encapsulated SN extract prompted a reduction in tumor weight and volume and, subsequently, boosted the survival chances up to 85%. The results of the current study supported the use of cyclodextrin-based nanosponges in delivering natural and synthetic anticancerous agents to reinforce respective stability and bioavailability.

## Data Availability

The original contributions presented in the study are included in the article/Supplementary Material; further inquiries can be directed to the corresponding authors.

## References

[B1] Aggarwal G. Nagpal M. Kaur G. (2016). Development and comparison of nanosponge and niosome based gel for the topical delivery of tazarotene. Pharm. Nanotechnol. 4, 213–228. 10.2174/2211738504666160804154213 29052500

[B2] Ahmad R. (2019). Steroidal glycoalkaloids from Solanum nigrum target cytoskeletal proteins: an *in silico* analysis. PeerJ 7, e6012. 10.7717/peerj.6012 30627484 PMC6321755

[B3] Albano D. Benenati M. Bruno A. Bruno F. Calandri M. Caruso D. (2021). Imaging side effects and complications of chemotherapy and radiation therapy: a pictorial review from head to toe. Insights Imaging 12, 76–28. 10.1186/s13244-021-01017-2 34114094 PMC8192650

[B4] Al-Ghani R. Nirwani W. P. Novianti T. N. Sari A. G. P. (2022). *In silico* anti-inflammatory activity evaluation from usnea misaminensis through molecular docking approach. Chem. Mat. 1, 77–82. 10.56425/cma.v1i3.40

[B5] Allahyari S. Zahednezhad F. Khatami M. Hashemzadeh N. Zakeri-Milani P. Trotta F. (2021). Cyclodextrin nanosponges as potential anticancer drug delivery systems to be introduced into the market, compared with liposomes. J. Drug Deliv. Sci. Technol. 67, 102931. 10.1016/j.jddst.2021.102931

[B6] Ansari K. A. Vavia P. R. Trotta F. Cavalli R. (2011). Cyclodextrin-based nanosponges for delivery of resveratrol: *in vitro* characterisation, stability, cytotoxicity and permeation study. AAPS PharmSciTech 12, 279–286. 10.1208/s12249-011-9584-3 21240574 PMC3066340

[B7] Argenziano M. Gigliotti C. L. Clemente N. Boggio E. Ferrara B. Trotta F. (2020). Improvement in the anti-tumor efficacy of doxorubicin nanosponges in *in vitro* and in mice bearing breast tumor models. Cancers 12, 162. 10.3390/cancers12010162 31936526 PMC7016577

[B8] Asela I. Donoso-González O. Yutronic N. Sierpe R. J. P. (2021). β-Cyclodextrin-Based nanosponges functionalized with drugs and gold nanoparticles. Pharm 13, 513. 10.3390/pharmaceutics13040513 PMC8068376 33917938

[B9] Bolmal U. B. Manvi F. Kotha R. Palla S. S. Paladugu A. Reddy K. R. J. (2013). Recent advances in nanosponges as drug delivery system. Int. J. Nanotechnol. 6, 1934–1944. 10.37285/ijpsn.2013.6.1.3

[B10] Chamundeeswari M. Jeslin J. Verma M. L. (2019). Nanocarriers for drug delivery applications. Environ. Chem. Lett. 17, 849–865. 10.1007/s10311-018-00841-1

[B11] Churiyah C. Ningsih S. Firdayani F. J. (2020). The cytotoxic, apoptotic induction, and cell cycle arrest activities of solanum nigrum L. ethanolic extract on MCF-7 human breast cancer cell. Asia-Pac. J. Clin. Oncol. 21, 3735–3741. 10.31557/APJCP.2020.21.12.3735 PMC8046323 33369475

[B12] Daga M. de Graaf I. A. Argenziano M. Barranco A. S. M. Loeck M. Al-Adwi Y. (2020). Glutathione-responsive cyclodextrin-nanosponges as drug delivery systems for doxorubicin: evaluation of toxicity and transport mechanisms in the liver. Toxicol Vitro 65, 104800. 10.1016/j.tiv.2020.104800 32084521

[B13] da Rocha Neto A. C. da Rocha A. B. Maraschin M. Di Piero R. M. Almenar E. (2018). Factors affecting the entrapment efficiency of β-cyclodextrins and their effects on the formation of inclusion complexes containing essential oils. Food Hydrocoll. 77, 509–523. 10.1016/j.foodhyd.2017.10.029

[B14] De Anda-Flores Y. Carvajal-Millan E. Lizardi-Mendoza J. Rascon-Chu A. Martínez-López A. L. Marquez-Escalante J. (2020). Covalently cross-linked nanoparticles based on ferulated arabinoxylans recovered from a distiller’s dried grains byproduct. Processes 8, 691. 10.3390/pr8060691

[B15] De Groot C. O. Hsia J. E. Anzola J. V. Motamedi A. Yoon M. Wong Y. L. (2015). A cell biologist’s field guide to aurora kinase inhibitors. Front. Oncol. 5, 285. 10.3389/fonc.2015.00285 26732741 PMC4685510

[B16] Deo S. V. S. Sharma J. Kumar S. (2022). GLOBOCAN 2020 report on global cancer burden: challenges and opportunities for surgical oncologists. Ann. Surg. Oncol. 29, 6497–6500. 10.1245/s10434-022-12151-6 35838905

[B17] Dong C. Tang X. Xie Y. Zou Q. Yang X. Zhou H. (2016). The crystal structure of an inactive dimer of PDZ-binding kinase. Biochem., Biophys. Res. Commun. 476, 586–593. 10.1016/j.bbrc.2016.05.166 27262437

[B18] Dong Y. Hao L. Fang K. Han X. Yu H. Zhang J. (2021). A network pharmacology perspective for deciphering potential mechanisms of action of Solanum nigrum L. in bladder cancer. BMC Complement. Med. Ther. 21, 45. 10.1186/s12906-021-03215-3 33494738 PMC7836472

[B19] Dora C. P. Trotta F. Kushwah V. Devasari N. Singh C. Suresh S. (2016). Potential of erlotinib cyclodextrin nanosponge complex to enhance solubility, dissolution rate, *in vitro* cytotoxicity and oral bioavailability. Carbohydr. Polym. 137, 339–349. 10.1016/j.carbpol.2015.10.080 26686138

[B20] Erum A. Bashir S. Saghir S. Hina S. Batool A. Mahmood T. (2014). Arabinoxylan isolated from ispaghula husk: a better alternative to commercially available gelling agents. Asian J. Chem. 26, 8366–8370. 10.14233/ajchem.2014.17495

[B21] Ferrati S. Nicolov E. Bansal S. Hosali S. Landis M. Grattoni A. (2015). Docetaxel/2-hydroxypropyl β-cyclodextrin inclusion complex increases docetaxel solubility and release from a nanochannel drug delivery system. Curr. Drug Targets. 16, 1645–1649. 10.2174/138945011614151119125541 25706254

[B22] Fink J. (2021). Petroleum engineer's guide to oil field chemicals and fluids. Third Edition. Gulf Professional Publishing.

[B23] Fouad Y. A. Aanei C. (2017). Revisiting the hallmarks of cancer. Am. J. Cancer Res. 7, 1016–1036. 28560055 PMC5446472

[B24] Galvão J. Silva V. Ferreira S. França F. Santos D. Freitas L. (2015). β-cyclodextrin inclusion complexes containing Citrus sinensis (L.) Osbeck essential oil: an alternative to control *Aedes aegypti* larvae. Thermochim. Acta. 608, 14–19. 10.1016/j.tca.2015.04.001

[B25] Gangwar S. Akhtar M. S. (2022). Formulation and characterization *in vitro* release of topical nanogel from natural source in the management of psoriasis. J. Cardiovasc. Dis. Res. 1, 45–62. 10.31838/jcdr.2022.13.01.05

[B26] Gezici S. Şekeroğlu N. (2019). Current perspectives in the application of medicinal plants against cancer: novel therapeutic agents. Curr. Med. Chem. Anticancer Agents 19, 101–111. 10.2174/1871520619666181224121004 30582485

[B27] Gholibegloo E. Mortezazadeh T. Salehian F. Ramazani A. Amanlou M. Khoobi M. (2019). Improved curcumin loading, release, solubility and toxicity by tuning the molar ratio of cross-linker to β-cyclodextrin. Carbohydr. Polym. 213, 70–78. 10.1016/j.carbpol.2019.02.075 30879691

[B28] Guo R. Cheng Y. Ding D. Li X. Zhang L. Jiang X. (2011). Synthesis and antitumoral activity of gelatin/polyoxometalate hybrid nanoparticles. Macromol. Biosci. 11, 839–847. 10.1002/mabi.201000434 21416607

[B29] Huang H. T. Seo H. S. Zhang T. Wang Y. Jiang B. Li Q. (2017). MELK is not necessary for the proliferation of basal-like breast cancer cells. elife 6, e26693. 10.7554/eLife.26693 28926338 PMC5605198

[B30] Iqbal M. S. Akbar J. Hussain M. A. Saghir S. Sher M. (2011). Evaluation of hot-water extracted arabinoxylans from ispaghula seeds as drug carriers. Carbohydr. Polym. 83, 1218–1225. 10.1016/j.carbpol.2010.09.024

[B31] Khazaei Monfared Y. Mahmoudian M. Caldera F. Pedrazzo A. R. Zakeri-Milani P. Matencio A. (2023). Nisin delivery by nanosponges increases its anticancer activity against *in-vivo* melanoma model. J. Drug Deliv. Sci. Technol. 79, 104065. 10.1016/j.jddst.2022.104065

[B32] Kumar A. Rao R. (2021). Enhancing efficacy and safety of azelaic acid via encapsulation in cyclodextrin nanosponges: development, characterization and evaluation. Polym. Bull. 78, 5275–5302. 10.1007/s00289-020-03366-2

[B33] Kumar S. Trotta F. Rao R. (2018). Encapsulation of babchi oil in cyclodextrin-based nanosponges: physicochemical characterization, photodegradation, and *in vitro* cytotoxicity studies. Pharmaceutics 10, 169. 10.3390/pharmaceutics10040169 30261580 PMC6321157

[B34] Kumar S. P. Girija A. S. Priyadharsini J. V. (2020). Targeting NM23-H1-mediated inhibition of tumour metastasis in viral hepatitis with bioactive compounds from Ganoderma lucidum: a computational study. Indian J. Pharm. Sci. 82, 300–305. 10.36468/pharmaceutical-sciences.650

[B35] Langie S. A. Azqueta A. Collins A. R. (2015). The comet assay: past, present, and future. Front. Genet. 6, 266. 10.3389/fgene.2015.00266 26322077 PMC4534839

[B36] Lembo D. Trotta F. Cavalli R. (2018). Cyclodextrin-based nanosponges as vehicles for antiviral drugs: challenges and perspectives. Nanomedicine 13, 477–480. 10.2217/nnm-2017-0383 29376455

[B37] Li J. H. Li S. Y. Shen M. X. Qiu R. Z. Fan H. W. Li Y. B. (2021). Anti-tumor effects of Solanum nigrum L. extraction on C6 high-grade glioma. J. Ethnopharmacol. 274, 114034. 10.1016/j.jep.2021.114034 33746002

[B38] Li Y. Gu C. J. P. M. (2022). Degalactotigonin inhibits invasion and induce apoptosis by targeting TGF-β signalling in oral cancer cells. Pharmacogn. Mag. 18, 1202–1210. 10.4103/pm.pm_7_22

[B39] Liu L.-Y. Yang Y.-K. Wang J.-N. Ren J.-G. (2022). Steroidal alkaloids from Solanum nigrum and their cytotoxic activities. Phytochem 202, 113317. 10.1016/j.phytochem.2022.113317 35820506

[B40] Lu Y. Liu Y. Yang C. (2017). Evaluating *in vitro* DNA damage using comet assay. J. Vis. Exp. 128, e56450. 10.3791/56450 PMC5752397 29053680

[B41] Mady F. M. Mohamed Ibrahim S. R. (2018). Cyclodextrin-based nanosponge for improvement of solubility and oral bioavailability of Ellagic acid. Pak. J. Pharm. Sci. 31, 2069–2076. 30393214

[B42] Minelli R. Cavalli R. Ellis L. Pettazzoni P. Trotta F. Ciamporcero E. (2012). Nanosponge-encapsulated camptothecin exerts anti-tumor activity in human prostate cancer cells. Eur. J. Pharm. Sci. 47, 686–694. 10.1016/j.ejps.2012.08.003 22917641

[B43] Mousavi-Kouhi S. M. Beyk-Khormizi A. Mohammadzadeh V. Ashna M. Es-haghi A. Mashreghi M. (2022). Biological synthesis and characterization of gold nanoparticles using Verbascum speciosum Schrad. and cytotoxicity properties toward HepG2 cancer cell line. Res. Chem. Intermed. 48, 167–178. 10.1007/s11164-021-04600-w

[B44] Nawaz A. Jamal A. Arif A. Parveen Z. (2021). *In vitro* cytotoxic potential of Solanum nigrum against human cancer cell lines. Saudi J. Biol. Sci. 28, 4786–4792. 10.1016/j.sjbs.2021.05.004 34354467 PMC8324988

[B45] Orellana E. A. Kasinski A. L. s. (2016). Sulforhodamine B (SRB) assay in cell culture to investigate cell proliferation. Bio-protoc. 6, e1984. 10.21769/BioProtoc.1984 28573164 PMC5448418

[B46] Palanisamy C. P. Cui B. Zhang H. Panagal M. Paramasivam S. Chinnaiyan U. (2021). Anti-ovarian cancer potential of phytocompound and extract from South African medicinal plants and their role in the development of chemotherapeutic agents. Am. J. Cancer Res. 11, 1828–1844. 34094656 PMC8167668

[B47] Paul S. Chakraborty S. Mukherjee A. Kundu R. (2015). Evaluation of cytotoxicity and DNA damaging activity of three plant extracts on cervical cancer cell lines. Int. J. Pharm. Sci. Rev. Res. 31, 183–189.

[B48] Petitjean M. Aussant F. Vergara A. Isasi J. R. (2020). Solventless crosslinking of chitosan, xanthan, and locust bean gum networks functionalized with β-cyclodextrin. Gels 6, 51. 10.3390/gels6040051 33333946 PMC7768548

[B49] Priyadarsini R. V. Murugan R. S. Maitreyi S. Ramalingam K. Karunagaran D. Nagini S. (2010). The flavonoid quercetin induces cell cycle arrest and mitochondria-mediated apoptosis in human cervical cancer (HeLa) cells through p53 induction and NF-κB inhibition. Eur. J. Pharmacol. 649, 84–91. 10.1016/j.ejphar.2010.09.020 20858478

[B50] Pushpalatha R. Selvamuthukumar S. Kilimozhi D. (2018). Cross-linked, cyclodextrin-based nanosponges for curcumin delivery-Physicochemical characterization, drug release, stability and cytotoxicity. J. Drug Deliv. Sci. Technol. 45, 45–53. 10.1016/j.jddst.2018.03.004

[B51] Sekaran G. James J. V. Li X. P. (2020). A network pharmacology-based approach of traditional indian medicinal plant solanum nigrum linn against hepatocellular carcinoma. J. Adv. Sci. Res. 11.

[B52] Shakil M. S. Rana Z. Hanif M. Rosengren R. J. (2022). Key considerations when using the sulforhodamine B assay for screening novel anticancer agents. Anticancer Res. 33, 6–10. 10.1097/CAD.0000000000001131 34261912

[B53] Shi F. Wang C. Wang L. Song X. Yang H. Fu Q. (2019). Preparative isolation and purification of steroidal glycoalkaloid from the ripe berries of Solanum nigrum L. by preparative HPLC–MS and UHPLC–TOF-MS/MS and its anti-non-small cell lung tumors effects *in vitro* and *in vivo* . J. Sep. Sci. 42, 2471–2481. 10.1002/jssc.201801165 31012280

[B54] Shringirishi M. Mahor A. Gupta R. Prajapati S. K. Bansal K. Kesharwani P. (2017). Fabrication and characterization of nifedipine loaded β-cyclodextrin nanosponges: an *in vitro* and *in vivo* evaluation. J. Drug Deliv. Sci. 41, 344–350. 10.1016/j.jddst.2017.08.005

[B55] Singh N. P. McCoy M. T. Tice R. R. Schneider E. L. (1988). A simple technique for quantitation of low levels of DNA damage in individual cells. Exp. Cell Res. 175, 184–191. 10.1016/0014-4827(88)90265-0 3345800

[B56] Thomas S. Gunasangkaran G. Arumugam V. A. Muthukrishnan S. (2022). Synthesis and characterization of zinc oxide nanoparticles of Solanum nigrum and its anticancer activity via the induction of apoptosis in cervical cancer. Biol. Trace Elem. Res. 200, 2684–2697. 10.1007/s12011-021-02898-6 34448982

[B57] Tiwari K. Bhattacharya S. (2022). The ascension of nanosponges as a drug delivery carrier: preparation, characterization, and applications. J. Mater Sci. Mater Med. 33, 28–21. 10.1007/s10856-022-06652-9 35244808 PMC8897344

[B58] Torne S. Darandale S. Vavia P. Trotta F. Cavalli R. (2013). Cyclodextrin-based nanosponges: effective nanocarrier for Tamoxifen delivery. Pharm. Dev. Technol. 18, 619–625. 10.3109/10837450.2011.649855 22235935

[B59] Tuan Anh H. L. Tran P. T. Thao D. T. Trang D. T. Dang N. H. Van Cuong P. (2018). Degalactotigonin, a steroidal glycoside from Solanum nigrum, induces apoptosis and cell cycle arrest via inhibiting the EGFR signaling pathways in pancreatic cancer cells. Biomed. Res. Int. 2018, 3120972–3120979. 10.1155/2018/3120972 30643798 PMC6311251

[B60] Varan C. Anceschi A. Sevli S. Bruni N. Giraudo L. Bilgic E. (2020). Preparation and characterization of cyclodextrin nanosponges for organic toxic molecule removal. Int. J. Pharm. 585, 119485. 10.1016/j.ijpharm.2020.119485 32497732

[B61] Vichai V. Kirtikara K. (2006). Sulforhodamine B colorimetric assay for cytotoxicity screening. Nat. Protoc. 1, 1112–1116. 10.1038/nprot.2006.179 17406391

[B62] Vukic M. D. Vukovic N. L. Popovic S. L. Todorovic D. V. Djurdjevic P. M. Matic S. D. (2020). Effect of β-cyclodextrin encapsulation on cytotoxic activity of acetylshikonin against HCT-116 and MDA-MB-231 cancer cell lines. Saudi Pharm. J. 28, 136–146. 10.1016/j.jsps.2019.11.015 31920439 PMC6950963

[B63] Wang H. C. Chung P. J. Wu C. H. Lan K. P. Yang M. Y. Wang C. J. (2011). Solanum nigrum L. polyphenolic extract inhibits hepatocarcinoma cell growth by inducing G2/M phase arrest and apoptosis. J. Sci. Food Agric. 91, 178–185. 10.1002/jsfa.4170 20853273

[B64] Wang X. Luo Z. Xiao Z. (2014). Preparation, characterization, and thermal stability of β-cyclodextrin/soybean lecithin inclusion complex. Carbohydr. Polym. 101, 1027–1032. 10.1016/j.carbpol.2013.10.042 24299871

[B65] Xu R. (2008). Progress in nanoparticles characterization: sizing and zeta potential measurement. Particuology 6, 112–115. 10.1016/j.partic.2007.12.002

[B66] Yaşayan G. Şatıroğlu Sert B. Tatar E. Küçükgüzel İ. (2020). Fabrication and characterisation studies of cyclodextrin-based nanosponges for sulfamethoxazole delivery. J. Incl. Phenom. Macrocycl. Chem. 97, 175–186. 10.1007/s10847-020-01003-z

[B67] Yoshimaru T. Nakamura Y. Katagiri T. (2021). Functional genomics for breast cancer drug target discovery. Hum. Genet. 66, 927–935. 10.1038/s10038-021-00962-6 PMC8384626 34285339

