# Supplementary material for: Preparation and investigation of a novel combination of Solanum nigrum-loaded, arabinoxylan-cross-linked β-cyclodextrin nanosponges for the treatment of cancer: in vitro, in vivo, and in silico evaluation
Source: Front Pharmacol. 2023 Dec 6;14:1325498. doi: 10.3389/fphar.2023.1325498 (PMC10730681; doi:10.3389/fphar.2023.1325498)
Supplement: Supplementary file 1 [file DataSheet1.PDF]

Preparation and investigation of a novel combination of *Solanum nigrum* loaded arabinoxylan cross-linked β-cyclodextrin nanosponges for the treatment of cancer: *In vitro, in vivo* and *in silico* evaluation

Hamid Saeed Shah[1], Sumera Zaib[2],* Imtiaz Khan[3],* Mahmoud A. Sliem[4], Osama Alharbi[4], Mohammed Al-Ghorbani[4], Zobia Jawad[5], Kiran Shahzadi[2], Sajjad Awan[6]

[1]Institute of Pharmaceutical Sciences, University of Veterinary and Animal Sciences, Lahore 54000, Pakistan
[2]Department of Basic and Applied Chemistry, Faculty of Science and Technology, University of Central Punjab, Lahore 54590, Pakistan
[3]Department of Chemistry and Manchester Institute of Biotechnology, The University of Manchester, 131 Princess Street, Manchester MI 7DN, UK
[4]Department of Chemistry, Faculty of Science, Taibah University, Medinah, Saudi Arabia
[5]Ladywillingdon Hospital, King Edward Medical University, Lahore, Pakistan
[6]College of Pharmacy, University of Sargodha, Sargodha, Pakistan

***Correspondence:** sumera.zaib@ucp.edu.pk (S.Z.); kimtiaz@hotmail.co.uk (I.K.)

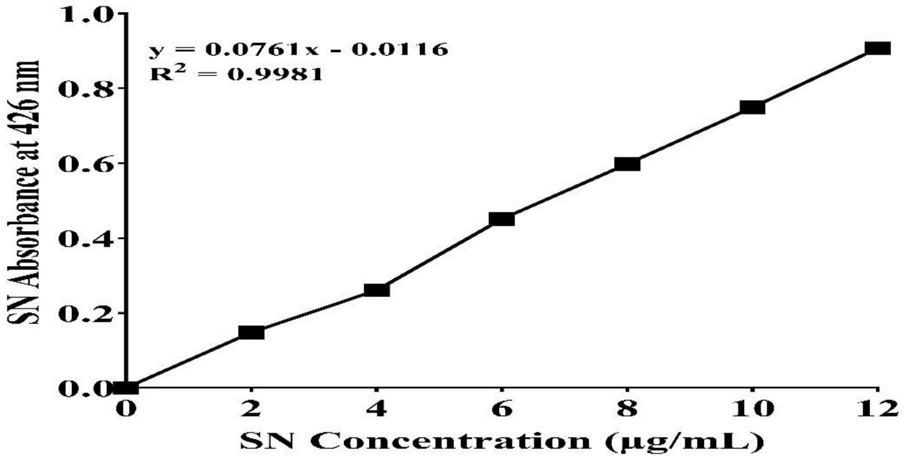

Figure S1. The standard curve for the SN extract was generated using a UV-Visible spectrophotometer.
